# Retinal Microvasculature and Conjunctival Vessel Alterations in Patients With Systemic Lupus Erythematosus—An Optical Coherence Tomography Angiography Study

**DOI:** 10.3389/fmed.2021.724283

**Published:** 2021-12-02

**Authors:** Wen-Qing Shi, Ting Han, Ren Liu, Qiang Xia, Tian Xu, Yan Wang, Shuang Cai, Shui-Lin Luo, Yi Shao, Rui Wu

**Affiliations:** ^1^Department of Immunology and Rheumatology, The First Affiliated Hospital of Nanchang University, Nanchang, China; ^2^Department of Ophthalmology, The First Affiliated Hospital of Nanchang University, Nanchang, China

**Keywords:** systemic lupus erythematosus, optical coherence tomography angiography, vessel density, conjunctival microvascular density, clinical indicators

## Abstract

**Purpose:** To evaluate the conjunctival and fundus retinal vessel density in patients with systemic lupus erythematosus (SLE) with optical coherence tomography angiography (OCTA), and to investigate the relationship between vessel density and clinical indicators.

**Methods:** Twelve patients with SLE (24 eyes) and 12 healthy controls (24 eyes) were recruited. OCTA was used to examine the superficial retina layer (SRL) and deep retina layer (DRL) in the macular retina and conjunctival capillary plexus of each eye. We calculated the density of the temporal conjunctival vessels, fundus microvascular (MIR), macrovascular (MAR) and total MIR(TMI) and compared the results in both groups. We used annular partitioning (C1–C6), hemispheric quadrants, and Early Treatment Diabetic Retinopathy Study partitioning (ETDRS) to analyze changes in the retinal vascular density. Correlation analysis was used to investigate the association between blood capillary density and clinical indicators.

**Results:** OCTA results showed significant differences in the conjunctival microvascular density (*p* < 0.001). There was no significant difference in MIR, TMI, and MAR in the superficial layers between the SLE and healthy group (*p* > 0.05). The DRL and DTMI (Deeper TMI) densities were decreased in the macular regions of SLE patients (*p* < 0.05). In the hemispheric segmentation analysis, the superficial MIR was significantly decreased in the IL (inferior left) region of the SLE patients (*p* < 0.05), and the deep MIR in the IR (inferior right) region was significantly reduced (*p* < 0.05). In the ETDRS partitioning analysis, the superficial MIR in the inferior, right, and left subdivisions was significantly decreased in the SLE patients (*p* < 0.05). In the circular segmentation analysis, the deep MIR in the C1 and C3 regions was significantly reduced in SLE patients (*p* < 0.05), while the superficial MIR density was decreased only in the C3 region (*p* < 0.05). The conjunctival vascular density was negatively correlated with the STMI (Superficial TMI) (*r* = −0.5107; *p* = 0.0108) and DTMI (*r* = −0.9418, *p* < 0.0001). There was no significant correlation between vascular density and SLEDAI-2k (Systemic Lupus Erythematosus Disease Activity Index−2000) (*P* > 0.05).

**Conclusion:** Clinically, patients with SLE and patients suspected of SLE should receive OCTA examination in a comprehensive eye examination to detect changes in ocular microcirculation at an early stage.

## Introduction

Systemic lupus erythematosus (SLE) is a chronic autoimmune disease that can cause changes in the whole body, including skin, joints, kidneys, and eyeballs. Approximately 33–35% of SLE patients have ocular disease (1). The incidence of ocular fundus lesions in SLE patients is about 15%. Previous studies have confirmed that visual impairment caused by SLE is often caused by retinal and optic neuropathy (2). Retinopathy can manifest as small intravascular hemorrhages, cotton wool spots, small arterial stenosis with capillary and venous expansion, and vascular curvature (3). SLE can lead to serious visual impairment or even blindness if left untreated. In some cases, the occurrence of ocular manifestations precedes the diagnosis of SLE, and if detected in time, a correct diagnosis of SLE is possible at early stages (4). A number of studies have suggested that SLE patients should receive eye examinations (5). Eye lesions are an important part of SLE disease activity and have become one of the important indicators of lupus activity according to the British Isles Lupus Assessment Group [BILAG (6)]. As a vascular disease, fundus examination and fundus fluorescence angiography (FFA) are the standard methods for posterior pathway evaluation of SLE patients. However, due to overlapping of superficial and deep capillary images, FFA lacks the ability to show differences in the major capillary networks, and traditional angiographic techniques cannot directly identify neovascularization and capture the entire retinal capillary system, leading to clinical misdiagnosis and missed diagnoses. In addition, some patients also suffer from adverse reactions such as allergy, vomiting, and nausea due to the contrast agent.

Optical coherence tomography angiography (OCTA) is a new imaging technique that has opened a new chapter in the diagnosis and treatment of fundus vascular disease. With high-resolution images, OCTA can reveal the supporting band capillary beds and their subtle changes that are not detected with fluorescence angiography (FA). OCTA uses motion contrast imaging combined with high-resolution volumetric blood flow information to create vascular images. A 3D vascular OCTA image is obtained by decoherence of the B-scan signal in two consecutive cross-sections, which divides the vessels into different layers for the purpose of blood flow detection. It is widely used in clinical practice and provides a non-invasive alternative for observing microvascular details (7–9). OCTA has been used in patients with Alzheimer's disease (AD) and it was found that the macular area thickness and central sulcus retinal thickness were decreased in both eyes in the AD group compared to normal controls (10). Fechut et al. (11) used OCTA in patients with multiple sclerosis (MS) and found significantly decreased retinal vascular density compared to healthy controls. The volume of the inner retinal structures and the superficial and deep vascular plexus densities were positively correlated. They suggested that alterations in choroidal vascularity may be related to disease activity in MS. Patients with coronary artery disease were found to have decreased overall and paracentral recess blood density in the deep retina of the macula using OCTA (12). No relevant studies have been conducted to study conjunctival and retinal vascular density in patients with SLE. The purpose of this study was to examine the retinal microvascular density and conjunctival density of SLE patients using OCTA and correlate the results with the clinical parameters of the patients.

## Materials and Methods

### Research Subjects

We recruited 12 SLE patients (24 eyes) at different stages of the disease and 12 healthy controls (24 eyes) for this study. All subjects were evaluated by retinal experts from the First Affiliated Hospital of Nanchang University between May 2020 and January 2021. We used the Systemic Lupus Erythematosus Disease Activity Index 2000 (SLEDAI-2 K) to assess disease activity. A score of 0–4 means basically no activity, a score of 5–9 means light activity, a score of 10–14 means moderate activity, and ≥15 means heavy activity (13). The Systemic Lupus Erythematosus International Cooperative Clinic (SLICC)/American College of Rheumatology (ACR) Injury Index (SDI) can be used to evaluate organ damage in SLE patients after a diagnosis of SLE (13). The control group consisted of healthy subjects, and an ophthalmologist from the medical center evaluated the absence of abnormalities in the eyes of these subjects through clinical examination and OCTA imaging. As shown in Table 1, non-experimental factors such as age, gender, and ESR were analyzed.

**Table 1:** Demographic characteristics and clinical findings of patients with SLE and HCs.

**Condition**	**HC**	**SLE**	***t***	***p***
Age (years)	33.17 ± 9.01	33.75 ± 9.08	−0.051	0.881
Sex (male/female)	1/11	1/11	–NA	–NA
Vision (R)	0.97 ± 0.07	0.75 ± 0.29	2.266	0.034
Vision (L)	0.82 ± 0.15	0.70 ± 0.25	1.329	0.197
IOP (R)	14.98 ± 1.39	16.22 ± 2.88	−1.289	0.211
IOP (L)	15.29 ± 1.73	16.23 ± 2.38	−0.854	0.402
ESR	15.42 ± 2.87	23.17 ± 21.91	−1.163	0.257
CRP	3.08 ± 2.14	6.96 ± 17.10	−0.746	0.464
Systolic pressure(mmHg)	129.08 ± 16.29	125.83 ± 6.71	0.612	0.547
Diastolic pressure (mmHg)	82.83 ± 6.68	78.08 ± 8.20	1.489	0.151
Duration (years)	–	4.33 ± 2.56	–NA	–NA
SLEDAI-2K	–	4.25 ± 2.83	–NA	–NA
SDI	–	5.25 ± 2.83	–NA	–NA

*HC, Healthy control; SLE, Systemic lupus erythematosus; IOP, Intraocular pressure; SLEDAI; 2K, Systemic Lupus Erythematosus Disease Activity Index−2000; SDI, Systemic Lupus Erythematosus International Cooperative Clinic/American College of Rheumatology Injury Index; ESR, Erythrocyte sedimentation rate; CRP, C-reactive protein; R, Right; L, Left*.

### Recruitment Criteria

All patients underwent clinical and ophthalmological evaluations at the first and last visits. Visual acuity and intraocular pressure were measured and all patients received corneal assessment and a fundus examination. The initial intraocular pressure range was 14–21 mmHg. The SLEDAI-2K was used to assess disease activity in SLE patients. Table 1 summarizes the general characteristics of the patients and the SLEDAI-2K scores of SLE patients.

### Exclusion Conditions

Smokers were excluded from the study. Except for the presence of SLE, patients with symptoms such as bleeding, vasculitis soft exudate, papilledema, or retinal detachment were excluded. Patients with hemorrhage and inflammation of the corneal conjunctiva of both eyes or eye diseases were excluded. Patients with systemic vascular diseases including diabetes, rheumatoid arthritis, polychondritis, and systemic hypertension were excluded. Pregnant or breastfeeding patients were excluded. Patients who were receiving long-term treatment for depression or anxiety were excluded. Patients with dilated pupils, intolerance of local anesthetic treatment within 6 months, or contraindications for eye surgery were excluded.

### Ethical Considerations

This study confirmed to the Declaration of Helsinki and had formal approval from the Medical Ethics Committee of the First Affiliated Hospital of Nanchang University. All the volunteers signed the informed consent forms and were given the opportunity to ask questions after learning about the purpose, content, and potential risks of this research.

### OCTA Imaging

**Figure 1:**
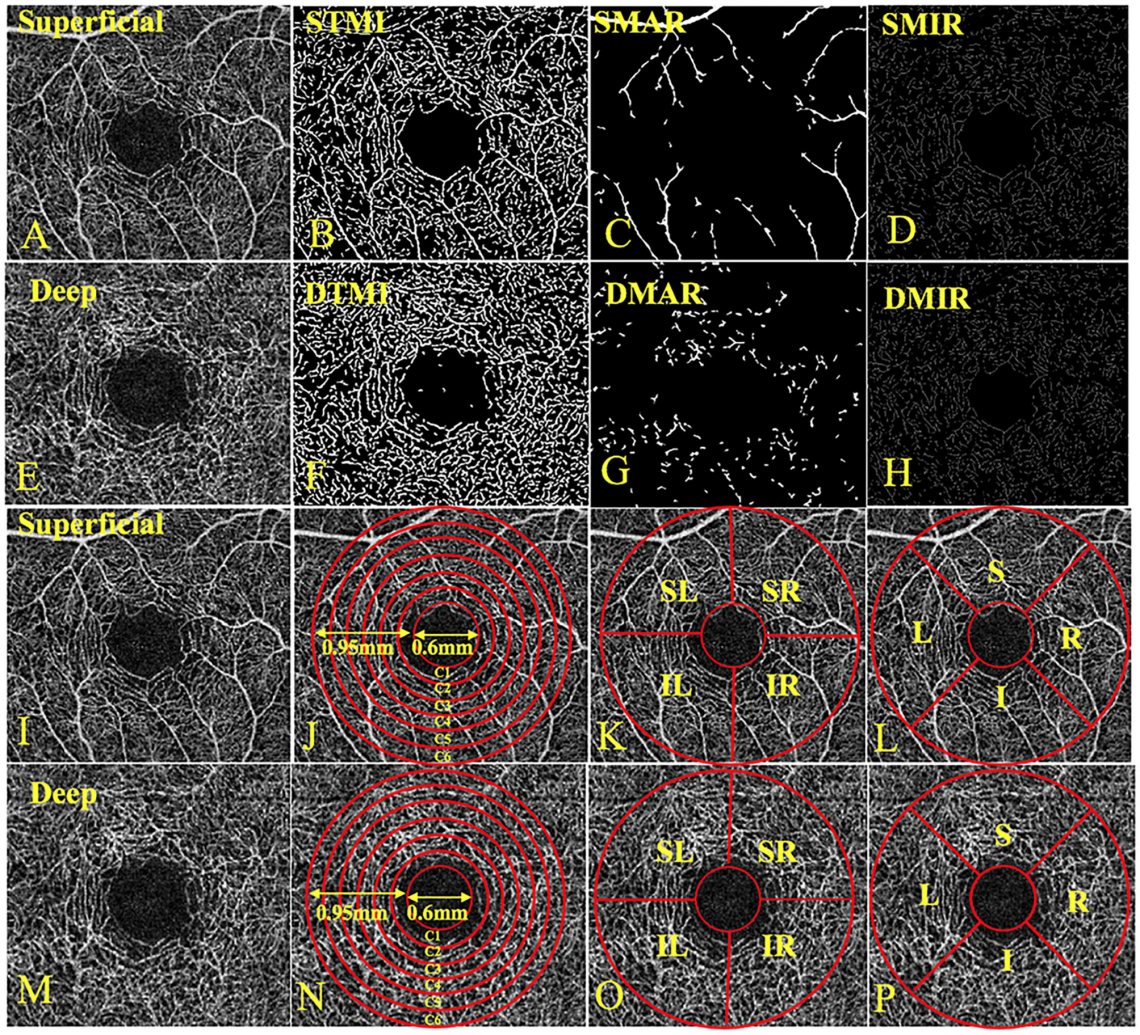
The 3 × 3-mm OCTA image of the retina and three division methods. **(A)** Superficial retinal vessel density map; **(B)** STMI, Superficial total microvascular; **(C)** SMAR, Superficial macrovascular; **(D)** SMIR, Superficial microvascular; **(E)** Deep retinal vessel density map; **(F)** DTMI, Deeper total microvascular; **(G)** DMAR, Deeper macrovascular; **(H)** DMIR, Deep microvascular; **(I)** Superficial retinal vessel density map; **(J)** Central annuli segmentation method in superficial retina layer; **(K)** Hemisphere segmentation method in superficial retina layer; **(L)** Early treatment of diabetic retinopathy study (ETDRS) method in superficial retina layer; **(M)** Deep retinal vessel density map; **(N)** Central annuli segmentation method in deep retina layer; **(O)** Hemisphere segmentation method in deep retina layer; **(P)** Early treatment of diabetic retinopathy study (ETDRS) method in deep retina layer. L, left; R, right; S, superior; I, inferior; IL, inferior left; IR, inferior right; SL, superior left; SR, superior right.

All OCTA examinations were completed by the same examiner using the Angio OCT optvue RTVue XR Avanti system. The scan speed was set to 70,000 A scans per second, the center wavelength was 840 nm, the bandwidth was 45 nm, the axial resolution was 5 mm, and the horizontal resolution rate was 22 μm. A B-scan (along the x-axis) in a 3 × 3-mm scan pattern with five repetitions of angiography was used to image at 216 raster positions (along the y-axis), focusing on the fovea, and the acquisition time was 3.9 s. We captured a 1080b scan (216y position × 5 position) at 270 frames per second (14). We obtained a 3 × 3 mm OCTA image through a series of four volume scans using two horizontal and two vertical rasters (933120a scans in total). The retinal capillary bed is artificially divided into two different physiological layers: the superficial retinal layer (SRL; the layer between the vitreous retinal interface and the front boundary of the ganglion cell layer) (Figures 1A–D,I–L) and the deep retinal layer (DRL; the inner plexiform layer, the layer between the inner boundary and the outer boundary of the outer plexiform layer) (Figures 1E–H,M,P). In the two stratifications, we analyzed the macrovascular (MAR) (Figures 1C,G), microvascular (MIR) (Figures 1D,H), and total microvascular (TMI; Figures 1B,F). Vessel density is defined as the ratio of the area of the perfused vessel to the measured area. In order to calculate the vessel density, a threshold algorithm was used to create a two-dimensional image of the SRL or DRL face. A value was determined for the image block and it was assigned to each pixel 1 (perfusion) or 0 (background). A similar length-based metric was used as a measure of blood vessel density. By taking the average of the skeletonized slab in the region of interest and considering the pixel distance (512 pixels per 3 mm), scaling results were used to calculate the density of blood vessels from the center of the macula to the edge of detection of the 3^*^3 mm image of the brightness gradient (14, 15). Then a series of customized segmentation algorithms were used to process the image, including inversion, balance, and removal of background noise and non-vascular structures, to generate a binary image. The skeleton image of a single capillary vessel with a diameter of >25 mm was obtained by removing small blood vessels. By convention, all subjects used the right eye. The data of the left eye were flipped to obtain a mirror image of the right eye. These figures were averaged and analyzed together with the right eye data.

**Figure 2:**
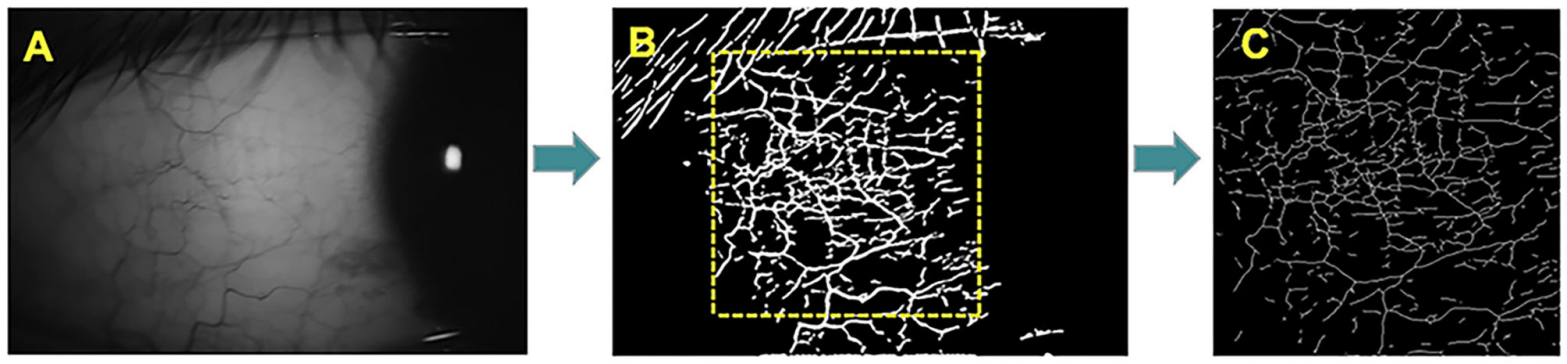
The OCTA image of the temporal conjunctiva. The microvascular network was extract in a field of 3 × 3-mm and analysis by the software. **(A)** Images of the patient's temporal conjunctiva. **(B)** OCTA scan of the temporal conjunctiva with microvascular density. **(C)** Microvascular density of 3 × 3 mm^2^ in region of interest.

Conjunctival microvascular images were acquired with the same parameters as used previously, with the lens adapter set at 2–4 cm from the subject's corneal surface for pre-ventilated contact between the adapter lens and the eye, and with manual focal adjustment and focus until the images were sharp (Figure 2).

### Macular Retinal Segmentation Method

The central annuli segmentation method included the following: After removing the avascular area (foveal diameter 0.6-mm), a circular area with a diameter of 0.6–2.5 mm was defined as an annular area with a bandwidth of 0.95 mm. The ring area was divided into six thin rings with a bandwidth of 0.16 mm each, named C1–C6. The hemisphere segmentation method included the following: The image was divided into four quadrants according to the horizontal and vertical diagonal lines, which were SR, SL, IL, and IR. The Early Treatment Diabetic Retinopathy Study (EDTRS) segmentation method included the following: The image was divided into four quadrants according to the diagonal of the two quadrants, R, S, L, and I in sequence. L = left; R = right; S = superior; I = inferior; IL = inferior left; IR = inferior right; SL = superior left; and SR = superior right (Figures 1D–F).

All scans were performed on all patients during the same time period from 12:00 to 2:00 to avoid possible diurnal variations.

### Statistical Analysis

The data were analyzed with statistical software (Statistica, Ver 7.1, StatSoft, Inc, Tulsa, OK and MedCalc software version 10, MedCalc Software, Mariakerke, Belgium). Data were presented as the mean ± SD. A *p*-value of <0.05 showed statistical significance. The receiver operating characteristic (ROC) curve was used to show the micro-vessel density of the superficial and deep retinal layers between the two groups.

## Results

### Macular and Conjunctival Vascular Density

**Figure 3:**
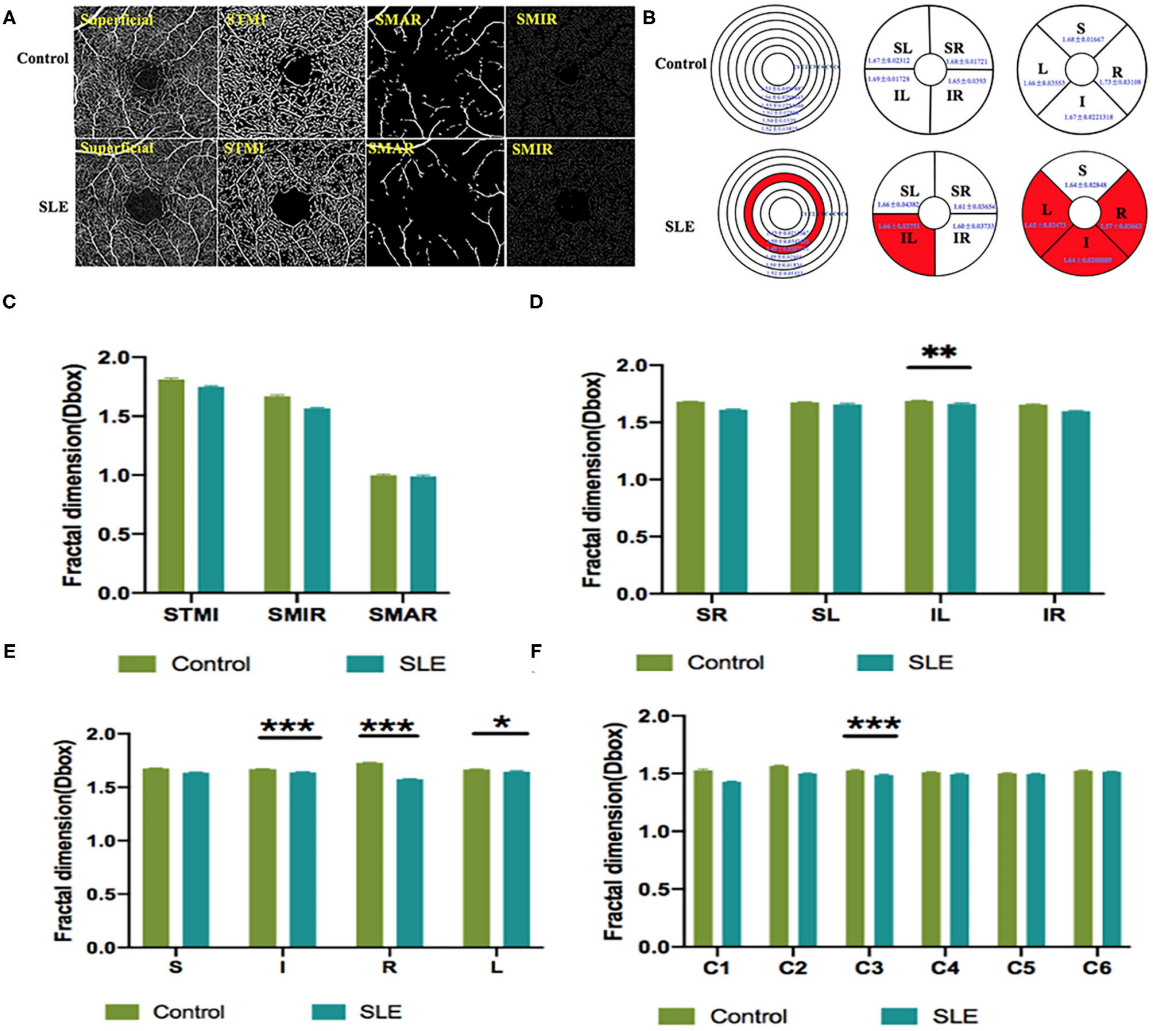
The OCTA images and vessel density analysis of superficial retinal layer of control and SLE groups. **(A)** Superficial retinal vessel density map. **(B)** Results of superficial retinal vascular density in different regions of normal group and SLE group (MD ± SD); **(C–F)** Results of superficial retinal vascular density analysis in normal and SLE groups. STMI, Superficial total microvascular; SMAR, Superficial macrovascular; SMIR, superficial microvascular; SLE, Systemic lupus erythematosus; L, left; R, right; S, superior; I, inferior; IL, inferior left; IR, inferior right; SL, superior left; SR, superior right. *P < 0.05; **P < 0.01; ***P < 0.001.

**Figure 4:**
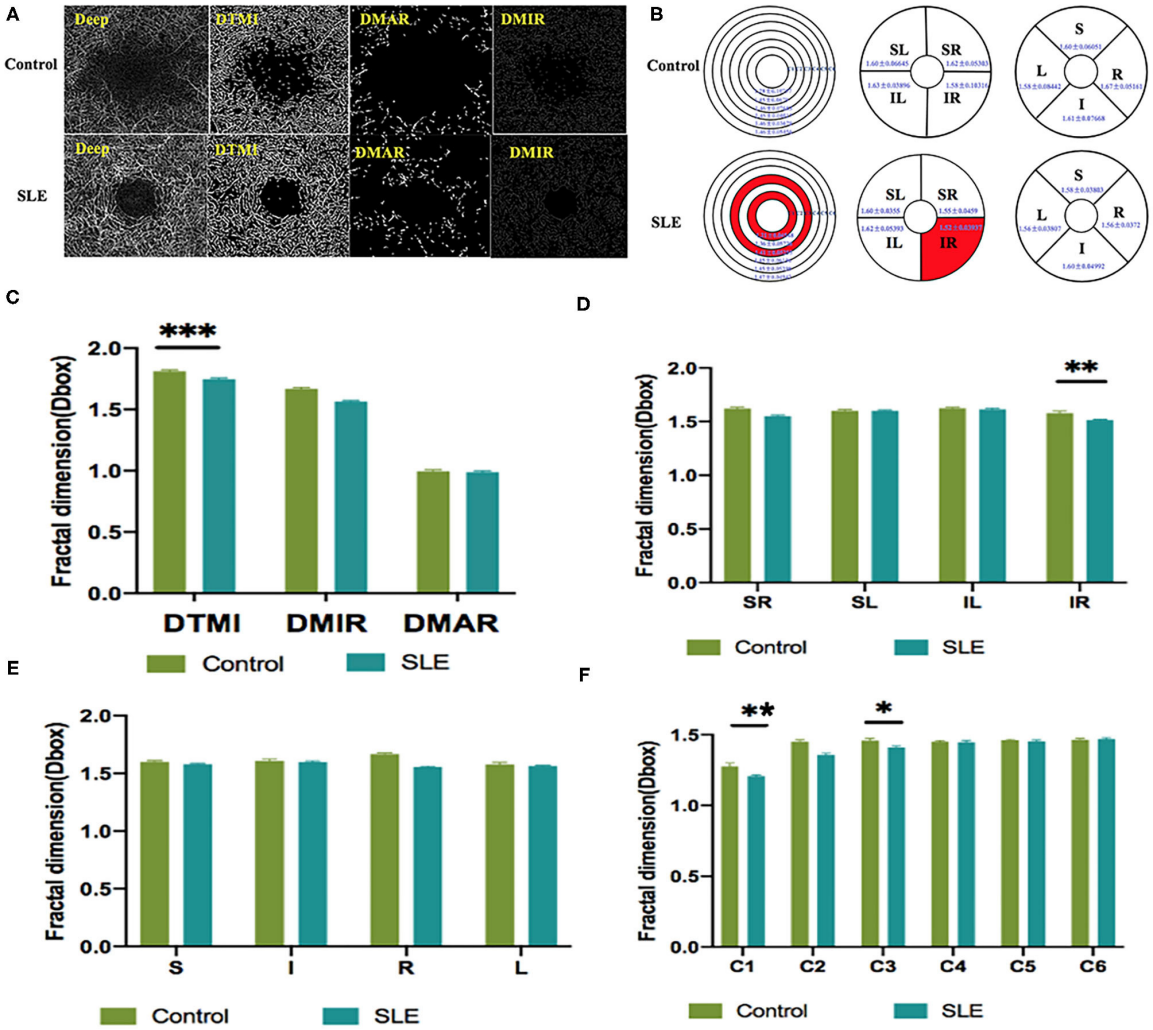
The OCTA images and vessel density analysis of deep retinal layer of control and SLE groups. **(A)** Deep retinal vessel density map. **(B)** Results of deep retinal vascular density in different regions of normal group and SLE group (MD ± SD); **(C–F)** Results of deep retinal vascular density analysis in normal and SLE groups. STMI, Superficial total microvascular; SMAR, Superficial macrovascular; SMIR, superficial microvascular; SLE, Systemic lupus erythematosus; L, left; R, right; S, superior; I, inferior; IL, inferior left; IR, inferior right; SL, superior left; SR, superior right. *P < 0.05; **P < 0.01; ***P < 0.001.

Macular vessel densities of the MIR, MAR, and TMI in the superficial (Figure 3) and deep (Figure 4) layers were compared. We observed no significant changes in SMIR, STMI, and SMAR in the superficial layers of SLE patients compared to the normal group (*P* > 0.05; Figure 3A). The DTMI density was significantly lower in SLE patients (*P* < 0.05; Figure 4A). Using the hemispheric segmentation method, we found that the IL region of superficial retinal vessels (*P* < 0.05; Figure 3B) and the IR region of deep retinal capillaries were significantly lower compared with healthy controls (*P* < 0.05; Figure 4B). In the ETDRS segmentation method, SMIR was significantly lower in the I.R.L region compared with healthy controls (*P* < 0.05; Figure 3C), while DMIR density showed no significant change in the quadrant (*P* > 0.05; Figure 4C). By comparing the micro-vessel density with the ring segmentation method, we found a significant decrease in vessel density in the superficial C1 and C3 regions (*P* < 0.05; Figure 3D). For the deep retinal layers, a significant decrease in micro-vessel density occurred in the C3 region (*P* < 0.05; Figure 4D). Other subdivisions also showed a decreasing trend, but the difference was not statistically significant (*P* > 0.05, Figures 3, 4).

OCTA was used to measure the blood vessel density of the temporal conjunctiva in the normal control group and the SLE group (**Figure 6**). The results showed that the density of the temporal conjunctiva of the SLE group was significantly higher than that of the normal control group (*t* = −8.089; *P* = 0.001; **Figure 6**).

### ROC Analysis of Superficial and Deep Retinal Vessel Densities

**Figure 5:**
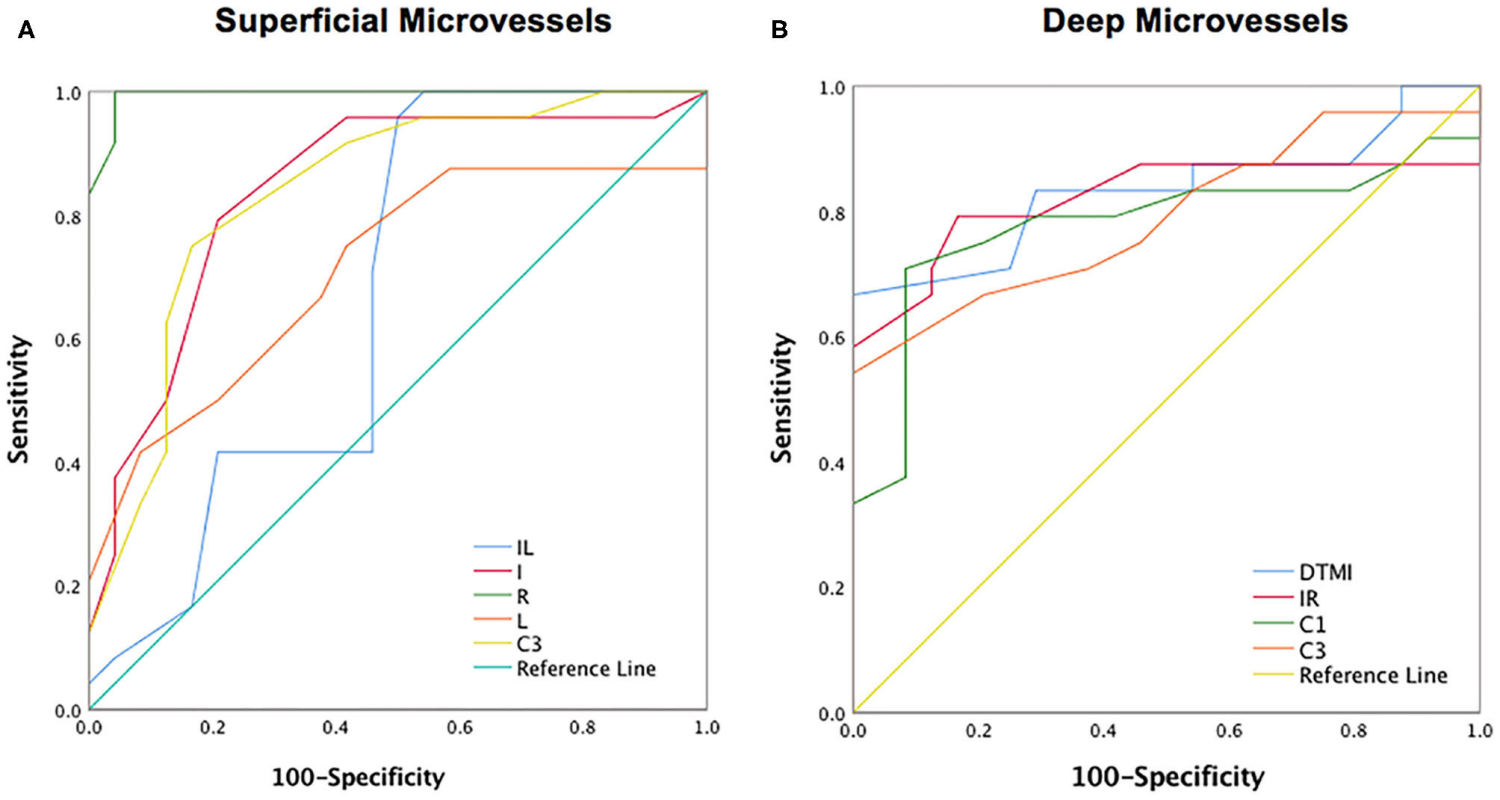
ROC curve analysis of sectorial, quadrantal and annular microvascular densities of retinal layer. **(A)** The ROC curve analysis of superficial retinal layer; **(B)** The ROC curve analysis of deep retinal layer. I, inferior; IL, inferior left; IR, inferior right; L, left; R, right; DTMI, Deeper total microvascular.

OCTA showed the specificity and sensitivity of retinal vessel densities to differentiate the SLE group from the healthy controls (Figure 5). In the superficial retinal layer, the IL, I, R, L, and C3 regions showed a significant difference in the SLE group (*P* < 0.05). Among them, the areas under the ROC curve of the R region of the superficial retinal density were 0.995 [95% confidence interval (CI) = 0.982–1], which indicated they had higher SLE diagnostic sensitivity for the superficial retinal density (Figure 5A). Meanwhile, the SLE group had the highest positive likelihood ratio for DTMI, with an area under the ROC curve of 0.832 (95% CI 0.708–0.956), and the lowest negative likelihood ratio for deep C1 retinal density. The area under the ROC curve was 0.779 (95% CI 0.633–0.925; Figure 5).

**Figure 6:**
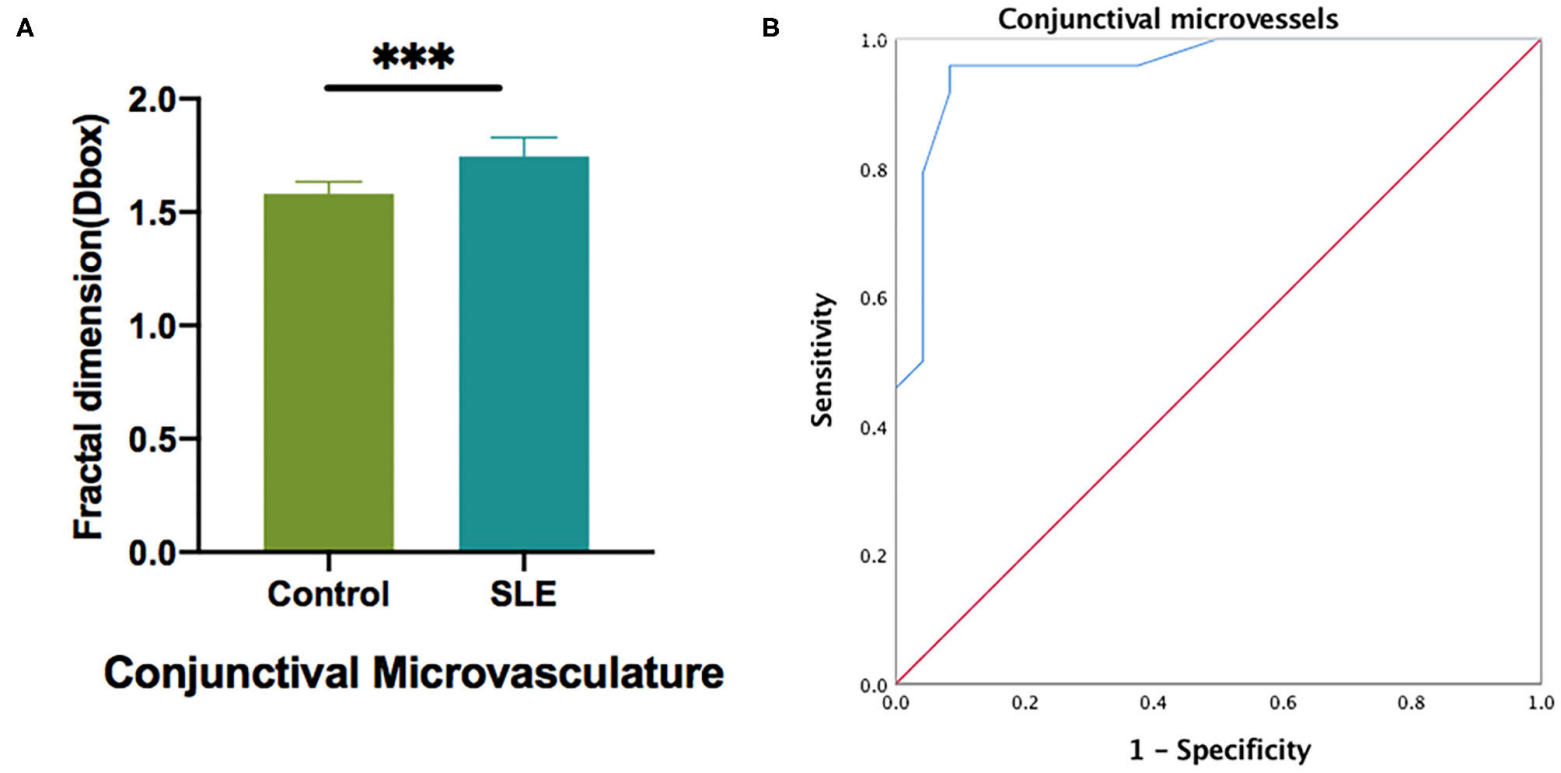
The conjunctival microvascular density analysis of control and SLE groups. **(A)** Statistic analysis of conjunctival microvascular in two groups; **(B)** The ROC curve analysis of conjunctival microvascular density. ***P < 0.001.

The ROC analysis of the conjunctival vessel density between the two groups showed that there was a significant difference in the conjunctival vessel density between the two groups. The area under the ROC curve was 0.957 [95% confidence interval (CI) = 0.902–1; Figure 6B].

### Relationship Between Macular Vascular Density and Conjunctival Vascular Density

**Figure 7:**
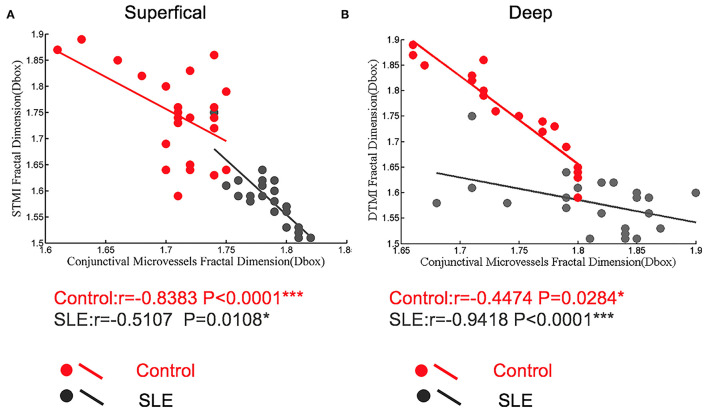
The correlation between conjunctiva microvascular density and retinal microvascular density in control and SLE patients. **(A)** In the superficial retinal layer; **(B)** In the deep retinal layer. *P < 0.05, ***P < 0.001.

We investigated the relationship between retinal vascular density and conjunctival vascular density. In the superficial retina of the SLE group, the correlation coefficient between the STMI area and temporal conjunctival vessel density was −0.5107 (*p* < 0.05; Figure 7A). Similarly, in the deep retinal layers, the correlation coefficient was −0.9418 (*p* < 0.0001; Figure 7B). In the normal controls, both retinal STMI and DTMI regions were significantly negatively correlated with conjunctival vessel density, with correlation coefficients of −0.8383 (*p* < 0.0001) and −0.4474 (*p* < 0.05), respectively (Figure 7).

## Discussion

As an immune system disease that affects the entire body, SLE can cause a variety of ocular complications, including keratoconjunctivitis, uveitis, retinal vascular abnormalities, and optic nerve and orbital damage (16). Typical retinal abnormalities of SLE include hemorrhage, cotton wool spots, twisted and expanded capillaries and veins, and stenosis and occlusion of arterial lumen (4). However, in SLE patients with early retinal lesions, subjective perception is not strong because the loss of vision is not obvious. By the time the loss of vision is obvious, the retinal lesions already worsened with the development of SLE lesions and treatment became relatively difficult. OCTA is used as a non-invasive, time-saving measure that provides detailed information on the perfusion of the ocular vascular network (17). It shows the microvascular condition without the use of a dye and is a safer screening test for patients. This study assessed the differences in retinal vascular density between SLE and health controls using OCTA. The results showed that, in the superficial capillaries of SLE patients, IL, I, R, L, and C3 regions had significantly lower densities compared to the healthy control group (*p* < 0.05), and in the deep retina layer, the DMTI, IR, C1, and C3 regions had significantly lower microvascular densities than healthy controls (*p* < 0.05; Figures 3, 4).

The pathological basis of SLE retinopathy is vasculitis (18). According to the data of previous animal experiments, the retinopathy of SLE patients is mainly caused by an abnormal retinal microvascular that supplies the nerve fiber layer (19). There are inflammatory cells, immunoglobulins, and complement deposited in the blood vessels of the retina, which is related to retinal vascular hypoperfusion (20). Early retinal vasculopathy may present with mucinous edema, and as the disease progresses, fibrin-like degeneration, necrosis, and thrombosis of small and medium-sized vascular connective tissue occur, resulting in hemorrhage and local ischemia and hypoxia (20). It has been suggested that severe vascular occlusion retinopathy is characterized by small artery occlusion and diffuse capillary non-perfusion (21). Our research found that the decrease in the density of IL, I, R, L, C3 in the superficial retina may be a precursor to obstructive vascular disease. From an anatomical point of view, the superficial retina and deep capillaries belong to two different topographical tissue structures (22). Birol et al. (23) found that, under normal conditions, the retinal circulation provides 15% of the oxygen supply to the inner retina during dark adaptation and only a small amount during light adaptation. In contrast, the outer retinal layer has relatively high circulatory requirements, and the DRL, which is critical for providing the metabolic needs of highly differentiated photoreceptors in the outer plexiform layer, may have a watershed area of supply that is vulnerable to ischemic damage in the retina, resulting in disruption of deep retinal structures (24–26). And previous studies have described that DRL is more prone to progressive obstruction than SRL and is attributed to hemodynamic dysfunction or impaired interactions between neurons, glial cells, and vascular cells (27). This is consistent with our finding that deep retinal microvessel density was significantly abnormal in SLE patients compared to the normal group (*P* < 0.05). The retinal vasculature is one of the vessels that can be directly observed in SLE patients, and its performance often reflects the degree of systemic vascular damage as well. SLE disease activity index is considered a strong predictor of damage and necrosis in this disease (28). Shaimaa et al. (29) found a negative correlation between SLICC and patients' deep capillary densities, while a direct correlation between SLICC and SLEDAI-2K and patients' vascular densities was not found in this study.

The conjunctival microcirculation is superficial, with clear images and easy to observe, thus the examination of bulbar conjunctival microcirculation has become a window to observe the microcirculation of systemic diseases. The conjunctival microcirculation is a component of the ocular microcirculation, which makes it even more relevant for the observation of ocular diseases. Currently, conjunctival microcirculation has been used to evaluate a variety of systemic diseases such as sickle cell disease (30), Alzheimer's disease (31), cardiopulmonary resuscitation (32), and cerebral perfusion (33). Conjunctival microcirculation has been associated with a variety of ophthalmology-related diseases such as hypertension-related eye disease, diabetes-related eye disease, and dry eye disease (34, 35). In this study, we found abnormal conjunctival vascular circulation in SLE patients. As a result of vascular inflammation and immune-mediated stimulation in SLE patients, the number or function of conjunctival cupped cells may be reduced, resulting in a decrease in mucin secretion (36). Acute inflammation is often accompanied by increased tear reflexes and blinking, while chronic inflammation may result in decreased conjunctival perception and reflex activity, and compensatory vasodilation leading to increased blood flow density (36). Previous studies have examined the ocular hemodynamic characteristics of patients with SLE using Color Doppler Flow Imaging (CDFI). The results of the study showed that the central retinal artery and posterior ciliary artery peak flow velocity during systole and the end diastolic flow velocities were significantly slower in the SLE group compared with the control group (37). The entire eye is supplied by the central retinal artery, except for the inner retina and part of the optic nerve. The rest of the eye is supplied by the posterior ciliary artery (38). The short posterior ciliary artery supplies the outer four layers of the retina, the macula, and the optic disc, which is consistent with our findings of decreased DTMI density. Pichi et al. (39) found increased blood flow in the iris vessels of SLE patients using OCTA when they examined SLE patients without ocular disease. In our study, we found a significant increase in conjunctival vascular density in patients with SLE, and we calculated a negative correlation between deep retinal DTMI and conjunctival vascular densities in patients with SLE (−0.9418; *p* < 0.0001). The iris and some of the conjunctival nutritional needs are supplied by branches of the anterior ciliary artery. Therefore, we speculated that the increased vascular density in the patient's conjunctiva may be caused by a compensatory increase in perfusion of the anterior ciliary artery.

There are some limitations in this study. The sample size of SLE patients was relatively small, however, with the small sample size, macular microvascular density and conjunctival density were significantly altered in SLE patients compared to the normal group (*p* < 0.05). These observations may lead to further development of more sensitive blood flow markers to detect retinal and conjunctival blood flow changes in early patients. And in the present study we found no correlation between retinal and conjunctival vascular density and SLEDAI-2K and other clinical indicators in SLE patients. This may require further large-scale studies.

## Conclusion

The results of this OCTA study suggested that patients with SLE have reduced macular MIR density, which may lead to a compensatory increase in conjunctival vascular density. A comprehensive ophthalmologic examination should be performed clinically in patients with SLE and those suspected of having SLE. OCTA provides a non-invasive quantitative assessment of retinal vascular density in SLE and can be used for early detection of retinal circulation changes. This may prevent the development of severe ocular pathology or even loss of vision.

## Data Availability Statement

The raw data supporting the conclusions of this article will be made available by the authors, without undue reservation.

## Ethics Statement

This study confirmed to the Declaration of Helsinki and had formal approval from the Medical Ethics Committee of the First Affiliated Hospital of Nanchang University. All the volunteers signed the informed consent forms and were given the opportunity to ask questions after learning about the purpose, content, and potential risks of this research.

## Author Contributions

W-QS, TH, and RL performed the experiments and collected the data. TX, YW, and SC designed the current study. S-LL, RW, and YS given final approval of the version to be published. W-QS wrote the manuscript. All authors have made substantial contributions to this research, read, and approved the final manuscript.

## Funding

This work was supported by the Key Research Foundation of Jiangxi Province (No. 20203BBG73059, 20181BBG70004), Excellent Talents Development Project of Jiangxi Province (20192BCBL23020), Natural Science Foundation of Jiangxi Province (20181BAB205034), Grassroots Health Appropriate Technology Spark Promotion Plan Project of Jiangxi Province (No. 20188003), Health Development Planning Commission Science Foundation of Jiangxi Province (No. 20201032), Health Development Planning Commission Science TCM Foundation of Jiangxi Province (No. 2018A060), Science and Technology Department Project of Jiangxi Province (No. 20161BBG70183), and Health and Family Planning Commission of Jiangxi Province (No. 2017Z008).

## Conflict of Interest

The authors declare that the research was conducted in the absence of any commercial or financial relationships that could be construed as a potential conflict of interest.

## Publisher's Note

All claims expressed in this article are solely those of the authors and do not necessarily represent those of their affiliated organizations, or those of the publisher, the editors and the reviewers. Any product that may be evaluated in this article, or claim that may be made by its manufacturer, is not guaranteed or endorsed by the publisher.
